# Genome-wide DNA methylome and transcriptome changes induced by inorganic nanoparticles in human kidney cells after chronic exposure

**DOI:** 10.1007/s10565-021-09680-3

**Published:** 2022-01-01

**Authors:** Andrea Soltysova, Patricia Begerova, Kristina Jakic, Katarina Kozics, Monika Sramkova, Eckart Meese, Bozena Smolkova, Alena Gabelova

**Affiliations:** 1 Department of Molecular Biology, Faculty of Natural Sciences, Comenius University in Bratislava, Ilkovicova 6, 841 04 Bratislava, Slovakia; 2 Institute of Clinical and Translational Research, Biomedical Research Center, Slovak Academy of Sciences, Dubravska cesta 9, 845 05 Bratislava, Slovakia; 3 Cancer Research Institute, Biomedical Research Center, Slovak Academy of Sciences, Dubravska cesta 9, 845 05 Bratislava, Slovakia; 4 Institute of Human Genetics, Saarland University, Building 60, 66421 Homburg, Germany

**Keywords:** Inorganic nanoparticles, Human renal cells, Whole transcriptome analysis, Genome-wide methylome analysis, Epigenetic toxicity

## Abstract

The unique physicochemical properties make inorganic nanoparticles (INPs) an exciting tool in diagnosis and disease management. However, as INPs are relatively difficult to fully degrade and excrete, their unintended accumulation in the tissue might result in adverse health effects. Herein, we provide a methylome–transcriptome framework for chronic effects of INPs, commonly used in biomedical applications, in human kidney TH-1 cells. Renal clearance is one of the most important routes of nanoparticle excretion; therefore, a detailed evaluation of nanoparticle-mediated nephrotoxicity is an important task. Integrated analysis of methylome and transcriptome changes induced by INPs (PEG-AuNPs, Fe_3_O_4_NPs, SiO_2_NPs, and TiO_2_NPs) revealed significantly deregulated genes with functional classification in immune response, DNA damage, and cancer-related pathways. Although most deregulated genes were unique to individual INPs, a relatively high proportion of them encoded the transcription factors. Interestingly, *FOS* hypermethylation inversely correlating with gene expression was associated with all INPs exposures. Our study emphasizes the need for a more comprehensive investigation of INPs’ biological safety, especially after chronic exposure.

**Graphical abstract:**

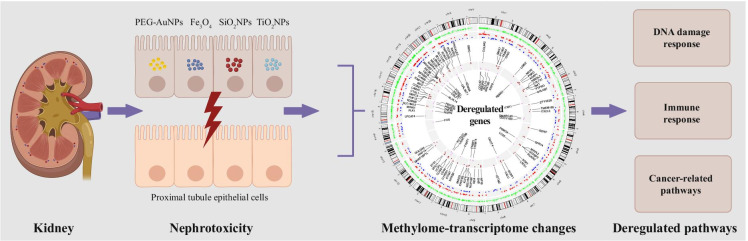

**Supplementary Information:**

The online version contains supplementary material available at 10.1007/s10565-021-09680-3.

## Introduction

Inorganic nanoparticles (INPs) have been widely used for a plethora of biomedical applications due to their unique physicochemical properties (e.g., magnetic, thermal, optical, or antibacterial). They provide more accurate imaging, diagnosis, innovative strategies for disease therapy *via* multimodal surface functional modifications and offer advanced solutions for regenerative medicine (Bayda et al. 2018). Integrating therapeutic and diagnostic properties in a single nanoscale platform (called theranostics) makes INPs an exciting tool in disease management. Gold nanoparticles (AuNPs) and magnetic iron oxide nanoparticles (NPs), mainly magnetite – Fe_3_O_4_NPs and maghemite – Fe_2_O_3_NPs, are promising contrast agents (e.g., magnetic resonance imaging, MRI, positron emission tomography, PET), heating mediators in hyperthermia-based cancer therapy, and nanovectors for targeted drug/gene delivery as well as molecular biosensors (Dadfar et al. 2019; Singh et al. 2018). Nowadays, silica NPs (SiO_2_NPs), particularly mesoporous silica NPs, are at the center of intensive research as a prospective drug delivery system. Large specific surface area and adjustable pore size allow loading of various therapeutic agents while protecting them from premature release and degradation in the body (Jafari et al. 2019). Titanium dioxide NPs (TiO_2_NPs) are becoming an important component in regenerative medicine as reinforcement material or as coatings improving osseointegration for the implants and as emerging antimicrobial agents (Jafari et al. 2020). Several INPs are currently being utilized in clinical practice, and many others are in clinical trials.

The biocompatibility and low toxicity are a prerequisite for biomedical applications of nanomaterials. Compared to soft nanomaterials (e.g., liposomes, dendrimers, organic polymers), INPs are relatively difficult to degrade completely and eliminate from the body; therefore, after their therapeutic or diagnostic use, they accumulate in the organism. The unintended long-term retention in the organs/tissues raises concerns about their safety. Despite intensive research, a conclusive understanding of the possible harmful health effects of these NPs has not been reached yet. Most in vitro and in vivo studies investigating the biosafety of INPs have focused primarily on their toxicity at cellular and genetic levels, their immunotoxic and inflammatory potential, and pathological effects, mainly after short-term (24–72 h) exposure (Dusinska et al. 2017). However, a growing body of evidence indicates the critical role of the deregulation of epigenetic regulatory mechanisms (Buocikova et al. 2020) in the pathogenesis of various complex human diseases, including cancer. Although the ability of heavy metals to induce aberrant epigenetic changes was repeatedly confirmed (Ray et al. 2014), the possible epigenetic toxicity of their nanoscale counterparts is still not satisfactorily explored. Considering the benefit of NPs, especially in the context of nanomedicine, their potential mid- or long-term adverse effects on human health require to be comprehensively investigated.

DNA methylation, a complex molecular mechanism regulating gene expression, is one of the most frequently studied epigenetic modifications. Hypermethylation of gene regulatory sequences (promoters or enhancers) often correlates with down-regulation of gene expression. On the contrary, gene-body methylation, frequently occurring (80–90%) in mammalian genomes, plays a vital role in preventing spurious transcription initiation and allowing efficient transcriptional elongation (Neri et al. 2017). DNA methylation alterations have been detected in different cell types exposed to several INPs and carbon-based NPs (reviewed in (Pogribna and Hammons 2021)). *In* vivo, it has been found after exposure to AuNPs, single-walled and multiwalled carbon nanotubes (Tabish et al. 2017), TiO_2_NPs (Ma et al. 2019), and copper NPs (Ognik et al. 2019). Recently, whole-genome DNA methylation changes have been identified in the blood of nanomaterial-handling workers exposed to metal oxide NPs (Liou et al. 2017) and those occupationally exposed during the nanocomposite producing processes (welding, machining) (Rossnerova et al. 2020).

Our study aims to investigate the capacity of INPs to affect the epigenome of the human renal epithelial TH-1 cells after chronic (7 days) exposure. This immortalized non-tumorigenic kidney cell line represents a suitable surrogate in vitro model of human renal proximal tubule cells. The kidneys are the primary organ for detoxification and excretion of toxicants from the body, and proximal tubule cells are particularly susceptible to xenobiotics, including NPs. Despite this fact, the number of studies focusing on nanomaterial-induced nephrotoxicity is limited. Recently, we have shown that none of the studied INPs (i.e., AuNPs coated with polyethylene glycol – PEG-AuNPs, Fe_3_O_4_NPs, SiO_2_NPs, and TiO_2_NPs), currently used in various biomedical applications, induces either DNA strand breaks or oxidative DNA damage in TH-1 cells after short-term (3 h and 24 h) exposure even at high concentrations (Sramkova et al. 2019). To better understand the potential nanobiointeractions at the molecular level, TH-1 cells were exposed to a subcytotoxic (2.2 μg/ml) concentration of INPs for 7 days without cell subculturing. This type of cell treatment is more physiologically relevant to in vivo situations than cell subculturing and repeated treatment. Genome-wide DNA methylation and transcriptome analysis were performed to comprehensively assess the biosafety of these INPs. To our best knowledge, data dealing with the epigenetic toxicity of INPs in renal cells are entirely lacking.

## Materials and methods

### Chemicals

Culture media, fetal bovine serum (FBS), antibiotics, and other chemicals used for cell cultivation were purchased from GIBCO (Gaithersburg, USA). All other chemicals and solvents from commercial suppliers were of analytical grade.

### Inorganic nanoparticles

**Fig. 1:**
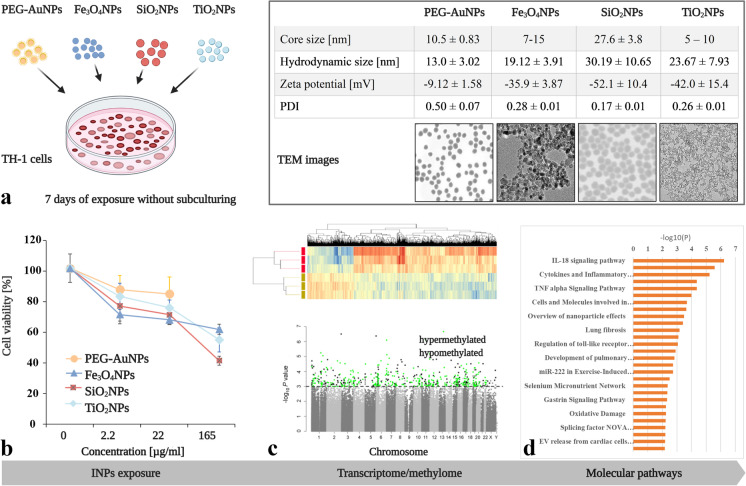
Experimental design and characterization of the INPs. Physico-chemical properties and transmission electron microscopy (TEM) images of individual INPs are shown in the table (top right). TH-1 cells were exposed for 7 days to different concentrations of four INPs (**a**). All INPs induced a dose-dependent decrease in cell viability after 7 days of exposure (**b**). A sub-cytotoxic concentration of 2.2 μg/ml was selected for further whole-genome analyses (methylome and transcriptome) (**c**). Altered metabolic pathways were assessed using pathway analysis (**d**)

All INPs used in the present study, namely PEG-AuNPs, Fe_3_O_4_NPs, SiO_2_NPs, and TiO_2_NPs, were kindly provided by Prof. Victor F. Puntes (Institute of Nanoscience and Nanotechnology, Barcelona, Spain). The dispersant used for Fe_3_O_4_NPs and TiO_2_NPs was TMAOH (tetramethylammonium hydroxide, 10 mM). PEG-AuNPs and SiO_2_NPs were kept in Milli-Q water. All NPs were characterized in-depth by different physical and chemical methods. The basic physicochemical characteristics of individual INPs in the stock solution are shown in Fig. 1 (table). The concentration of stock solution of PEG-AuNPs was 0.396 mg Au/ml, Fe_3_O_4_NPs was 11.7 mg Fe_3_O_4_/ml, SiO_2_NPs was 10.15 mg SiO_2_/ml, and TiO_2_NPs was 5 mg TiO_2_/ml. All INPs were stable in stock solution and did not form aggregates. The behavior of these INPs in culture medium (particle size distribution and colloidal stability) has already been published (Sramkova et al. 2019).

### Human cell line

The human renal proximal tubule epithelial TH-1 cell line was purchased from Kerafast Inc. (Boston, USA). The cells were cultivated in Dulbecco’s Modified Eagle Medium (DMEM) with high glucose (4.5 g/l) supplemented with 10% FBS and antibiotics (penicillin 100 U/ml, streptomycin, 100 μg/ml) at 37°C in a humidified atmosphere of 5% CO_2_.

### Cell treatment

Exponentially growing TH-1 cells were exposed to INPs for 7 days. The working concentrations were prepared freshly before the exposure according to the protocol provided by Prof. Victor F. Puntes. In brief, 1 part of the INPs in the stock solution was initially mixed with 1 part of FBS (dilution 1:1), and then a culture medium with FBS (9 parts) was added. Further dilutions were prepared from this dispersion of INPs in the culture medium. As a control, we used TH-1 cells kept in a culture medium for 7 days. The solvents/dispersants (TMAOH, Milli-Q water) were also tested for potential cytotoxic effects after 7 days of treatment. Cells were exposed to solvents at the concentration they reached at the highest (165 μg/ml) INPs concentration. We did not include the solvent controls in our whole-genome analyses because they affected neither cell viability nor inhibited proliferation and induced apoptosis (Fig. S1). The treatment of the cells was finished by aspirating the medium and washing the cells twice with phosphate-buffered saline (PBS). Cells were processed immediately, and snap-frozen cell pellets were kept at – 80°C until molecular analysis.

### Cytotoxicity of INPs

Exponentially growing TH-1 cells seeded at a density of 2 × 10^4^/well were exposed to INPs, and solvents/dispersants, for 7 days. After treatment, cells were incubated with 100 μl of working solution of alamarBlue® (Invitrogen, USA) for 4 h according to the manufacturer’s protocol. The fluorescence (excitation 530 nm, emission 590 nm) in each well was measured on a microplate reader – POLARStar OPTIMA (BMG Labtech, Germany).

### DNA and RNA isolation

Cell pellets for RNA extraction (*n* = 3 independent biological replicates per type of INPs and control cells) were frozen in TRIzol® solution and stored at − 80°C until extraction. Total RNA was isolated using Direct-zol™ RNA Miniprep (Zymo Research, USA) according to the manufacturer’s instructions. RNA integrity number (RIN) was evaluated by capillary electrophoresis using Agilent RNA 6000 Nano Kit (Agilent Technologies, USA), and RNA quantity was measured using NanoDrop ND-2000 (Nanodrop Technologies, Inc., USA). RNA samples with RIN above 8 were used for gene expression analysis. DNA from controls and NP-exposed TH-1 cells (*n* = 3 independent biological replicates per each type of INPs and control cells) was extracted using Gentra Puregene Blood Kit (Qiagen, Hilden, Germany) following the manufacturer’s instructions. The DNA concentration, purity, and absorbance ratios were assessed by NanoDrop Spectrophotometer.

### Gene expression microarray assays

The 100 ng of total RNA was labeled using the Low Input Quick Amp Labeling Kit (Agilent Technologies, USA) according to the manufacturer’s instructions. Briefly, RNA was transcribed into cDNA using T7-primer and Affinity Script RNase Block Mix. All subsequent labeling reactions were performed using Cy3-dCTP to obtain labeled cRNA. Labeled cRNA was purified using GeneJET^TM^ RNA Purification Kit (Thermo Fisher Scientific, USA) to remove non-incorporated nucleotides. Subsequently, 600 ng of the labeled sample was fragmented by 30-min incubation at 60°C using Gene Expression Hybridization Kit (Agilent Technologies, USA) components. Samples were immediately applied onto SurePrint G3 Human Gene Expression 8x60K Microarray Slide (Agilent Technologies, USA) and hybridized 17 h at 65°C by rotating the slide at a speed of 10 rpm in hybridization oven (Agilent Technologies, USA). After hybridization, slides were washed (Gene Expression Wash Buffer Kit, Agilent Technologies, USA) and scanned at resolution 2 μm using SureScan Microarray Scanner (Agilent Technologies, USA).

### Methylated DNA immunoprecipitation array analysis

Methylated regions of the genome (controls and treated TH-1 cells) were immunoprecipitated using a 5-methylcytidine monoclonal antibody (Eurogentec, Belgium) following an Agilent microarray analysis of methylated DNA immunoprecipitation protocol. Immunoprecipitated DNA (using Cy5-dUTP) and non-immunoprecipitated DNA (using Cy3-dUTP) from the same sample were differently labeled using SureTag DNA Labeling Kit (Agilent Technologies, USA). Samples were purified using column purification (SureTag DNA Labeling Kit, Agilent Technologies, USA), mixed, and prepared for hybridization by incubation at 95°C for 3 min, followed by 37°C for 30 min using Oligo aCGH/ChIP-on-chip Hybridization Kit (Agilent Technologies, USA). The mix of labeled DNAs was then hybridized onto the Agilent custom methylation microarray 2 × 400 K, design ID 086060 (Agilent Technologies, USA), containing a combination of probes from designs ID 023795 and ID 014791. After 40 h hybridization at 67°C by rotating slide at speed 20 rpm in hybridization oven (Agilent Technologies, USA), slides were washed (Agilent Oligo aCGH/ChIP-on-Chip Wash Buffer Kit, Agilent Technologies, USA) and scanned at resolution 3 μm using SureScan Microarray Scanner (Agilent Technologies, USA).

### Image and data analysis

TIFF multiscan images from SureScan Microarray Scanner (Agilent Technologies, USA) were processed using Feature Extraction Software 11.5 (Agilent Technologies, USA). In this software also, the image processing was performed and acquired files with spot intensities for every microarray field (corresponding to one condition). The raw data underwent quality control, normalization, and statistical analysis in GeneSpring 14.9 GX software for gene expression analysis and Agilent Genomic Workbench 7.0.4.0 for DNA methylation analysis.

### DNA methylation and gene expression analysis

The differences in gene expression were analyzed comparing the control group vs. the appropriate condition evaluated separately (non-averaged) using a moderate *T* test (GeneSpring). Significant differences in gene expression between groups were considered when *p* < 0.05. A cut-off for fold-change values was not included based on previously published argumentation (Gliga et al. 2018), showing that low-dose NPs exposure might warrant important, but low fold changes in the gene expression.

Significantly different methylations were obtained by using an unpaired Student’s *T* test with a *p*-value cut-off *p* < 0.05, BATMAN algorithm, and the delta beta (Δβ) value, calculated as the difference of the average beta values of test and control samples. All probes differentially methylated (*p* < 0.05) were analyzed against a set of expression probes (*p* < 0.05). Primary findings were considered based on the localization of probes within the gene. Cluster analysis and volcano plots were performed using GeneSpring 14.9 GX for a selected set of genes. Circular plots were generated using the R/Shiny application, graphical interface shiny Circos.

A pathway analysis (GeneSpring) was performed to revealed molecular pathways significantly altered by the treatment (*p* < 0.05). Transcription factors (TFs) interaction network was created using FunRich, an open-access standalone functional enrichment analysis tool.

### Validation of FOS gene expression using qRT-PCR

For these experiments, we used the same total RNA as was used for microarray analysis. The total RNA was transcribed into cDNA using RevertAid First Strand cDNA Synthesis Kit (Thermo Fisher Scientific, USA) following the manufacturer's recommendations. The 20 μl qRT-PCR reaction contained HOT FIREPol® EvaGreen® qPCR Mix Plus (Solis BioDyne, Estonia) and 0.3 μM of appropriate primers. To obtain more accurate results, we selected three pairs of housekeeping genes (hypoxanthine phosphoribosyltransferase 1, *HPRT1*, succinate dehydrogenase complex flavoprotein subunit A, *SDHA* and TATA-box-binding protein *TBP*). Sequences of primers are listed in Table S1. The reaction conditions were set as follows: 95°C 10 min followed by 40 cycles of 95°C for 30 s, 61°C for 30 s, and 72°C for 30 s using Real-Time PCR Thermal Cycler qTOWER3 (Jena Analytics, Germany). Gene expression changes were calculated using the ΔΔCT method; results were averaged using all three independent housekeeping genes and three independent samples for every nanoparticle and control experiment. All qRT-PCR analyses for every sample were performed in triplicates.

## Results

### Cytotoxicity of INPs after long-term treatment

The viability of TH-1 cells after 7 days of exposure was assessed at three concentrations, 2.2, 22, and 165 μg/ml (corresponding to 1, 10, and 75 μg/cm^2^) in the case of Fe_3_O_4_NPs, SiO_2_NPs, and TiO_2_NPs (Fig. 1a). Regarding PEG-AuNPs, only concentrations 2.2 and 22 μg/ml were used due to the lower concentration of the stock solution. These concentrations were selected based on the recommendation of EU-funded NaNoReg and NanoTest projects (Yamani et al. 2017). All INPs induced a dose-dependent decrease in cell viability after 7 days of exposure (Fig. 1b).

PEG-AuNPs were the least cytotoxic when compared to Fe_3_O_4_NPs, SiO_2_NPs, and TiO_2_NPs. Based on the survival profile of INPs after 7 days of treatment, the concentration of 2.2 μg/ml was selected for subsequent whole-genome analyses. The viability of the exposed cells at this concentration ranged between 71 and 86%. The cell viability was investigated after 7 days of exposure to the solvent (Milli-Q water and TMAOH). Neither of the solvents significantly reduced the cell viability and proliferation and induced apoptosis at the tested concentration (Fig. S1).

### Nanoparticles induced whole-genome transcriptional changes

**Fig. 2:**
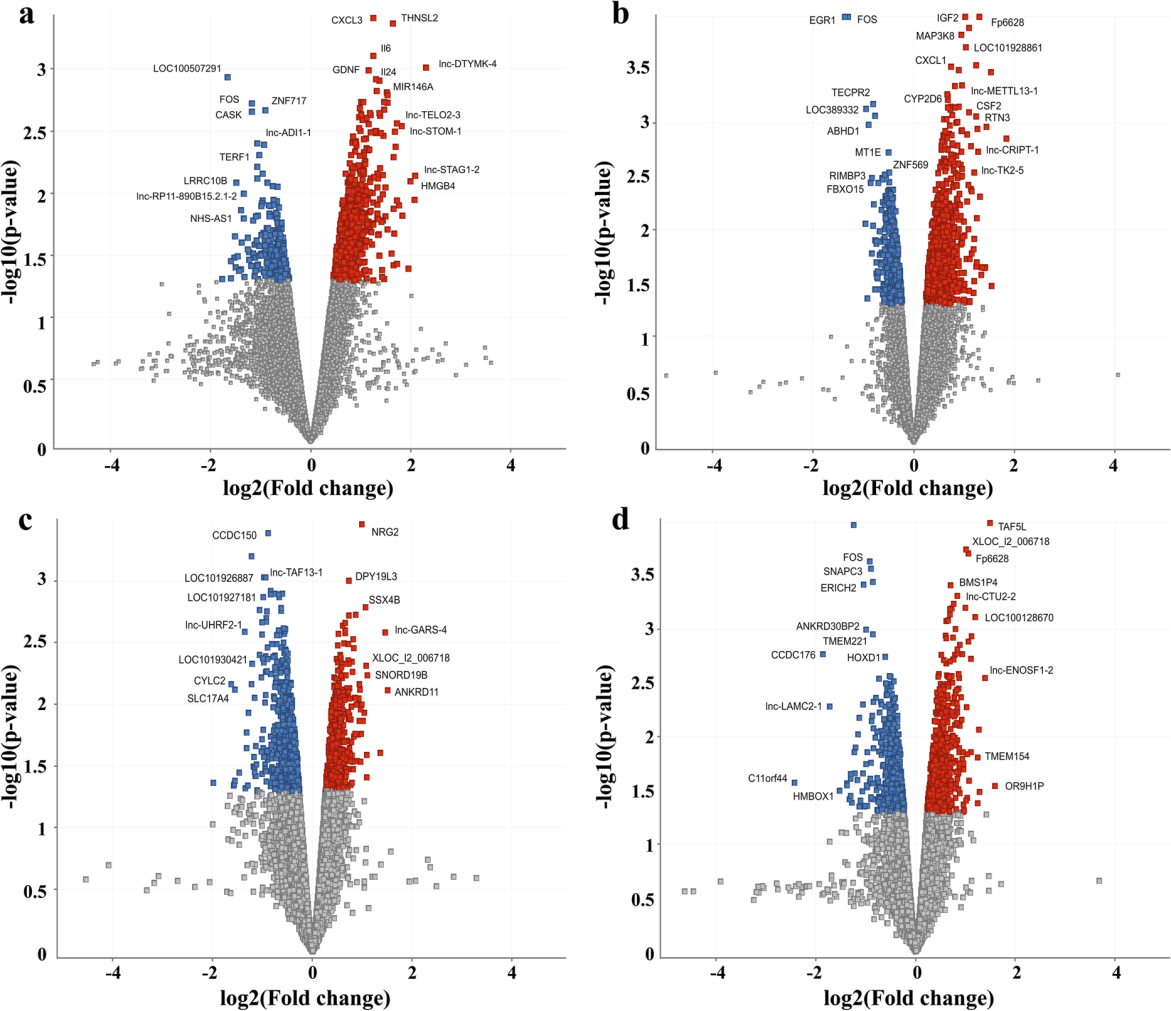
Volcano scatter plots showing the distribution -log10 (*p*-value) (*y*-axis) and log2 (fold change) (*x*-axis) of changes in mRNA expression induced by exposure to PEG-AuNPs (**a**), Fe_3_O_4_NPs (**b**), SiO_2_NPs (**c**), and TiO_2_NPs (**d**) compared to non-treated controls. In each plot, significantly up-regulated entities are highlighted by red, down-regulated by blue, the most significant transcripts are identified by gene abbreviation (if mapped). Non-significant findings are presented as grey dots

To better understand the nanobiointeractions induced by INPs at the molecular level, whole-genome transcriptome analysis was performed. It revealed significant deregulation of a high number of entities/genes after long-term exposure to individual INPs (Fig. 2, Table S2). However, changes higher than 2-fold were present only in a substantially lower number of them. The highest number of deregulated entities with at least 2-fold change was associated with PEG-AuNPs exposure (*n* = 190 corresponding to 185 genes), followed by SiO_2_NPs and TiO_2_NPs (*n* = 45 corresponding to 45 genes), and Fe_3_O_4_NPs (*n* = 43 corresponding to 43 genes). All significantly deregulated entities are listed in Table S3.

**Fig. 3:**
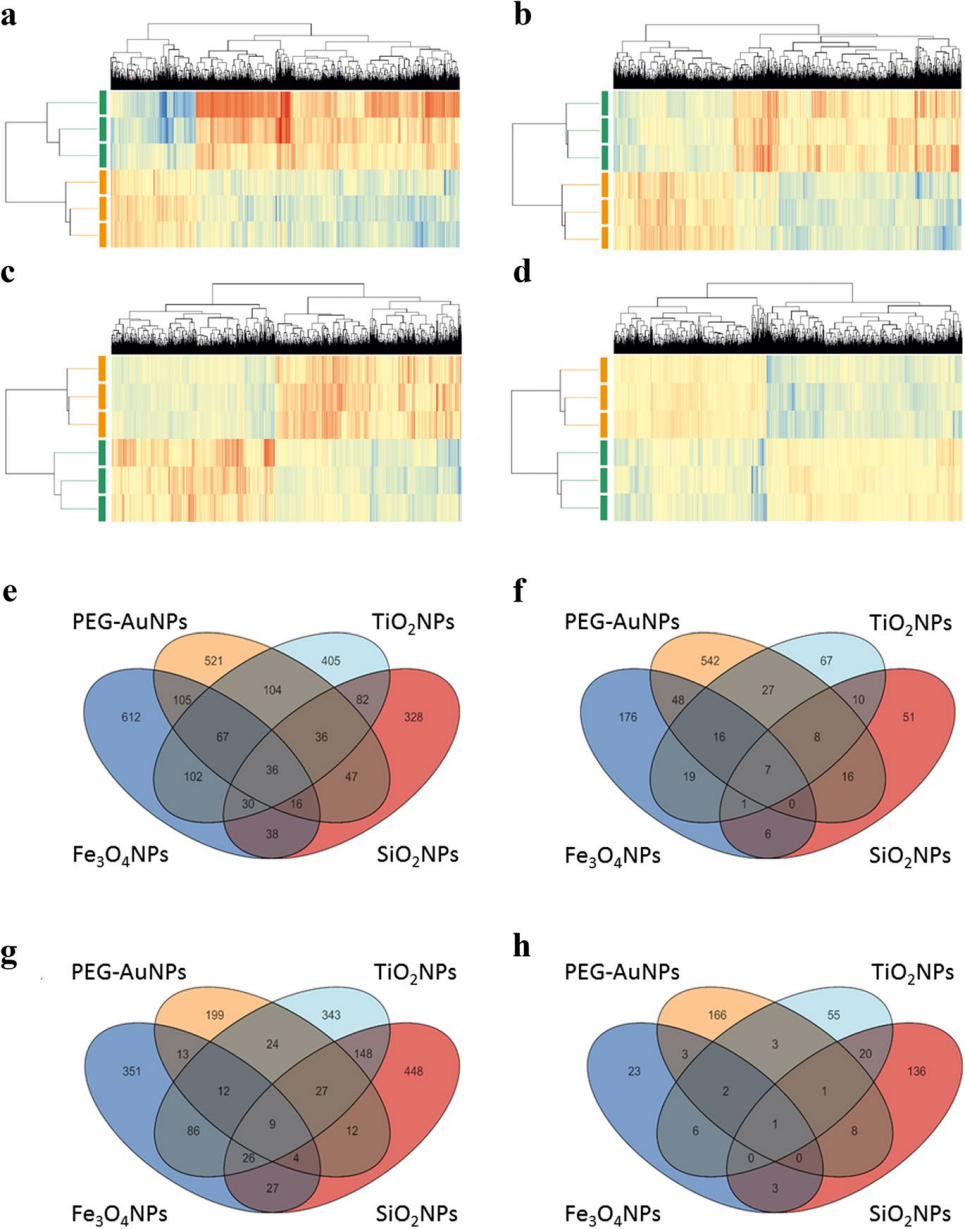
Heatmaps of significantly deregulated genes for individual exposure types (**a**–**d**) and Venn diagrams showing differentially expressed genes overlap between analyzed INPs (**e**–**h**). Cluster analysis of significantly changed transcripts for PEG-AuNPs (**a**), Fe_3_O_4_NPs (**b**), SiO_2_NPs (**c**), and TiO_2_NPs (**d**) exposed cells (marked by green) compared to non-treated controls (marked by orange). Three independent experiments are shown. Up-regulated entities are highlighted by red, down-regulated by blue, those which remain unchanged are yellow. Venn diagrams show overlapping up-regulated (**e**, **f**) and down-regulated (**g**, **h**) genes. On the left side (**e**, **g**) are shown all significantly deregulated genes, while those with higher than 1.5-fold change are shown on the right side (**f**, **h**) of the figure

Results of unsupervised hierarchical clustering of significantly altered gene expression induced by exposure to INPs are shown in Fig. 3a–d. Significantly deregulated genes formed distinct expression clusters, characteristic for individual INPs exposure. The numbers of deregulated genes by individual INP exposures are shown in Fig. 3e–h. While PEG-AuNPs and Fe_3_O_4_NPs exposures mainly resulted in up-regulation of gene expression, the 10 top-ranked genes were primarily down-regulated after SiO_2_NPs and TiO_2_NPs exposure (Table 1). In total, up-regulated genes covered 76%, 66%, 47%, and 56% of all deregulated genes for PEG-AuNPs, Fe_3_O_4_NPs, SiO_2_NPs, and TiO_2_NPs, respectively. Interestingly, among the highest-ranking genes prevalent were those coding for long non-coding RNAs (lncRNA). Their ratios among the top-ranked genes for PEG-AuNPs, Fe_3_O_4_NPs, SiO_2_NPs, and TiO_2_NPs were 50%, 30%, 30%, and 50%, respectively. Most of the deregulated genes were unique for every type of INPs (Table 2, Fig. 3e–h, Table S3); however, we identified 36 genes up-regulated and 9 down-regulated by the exposure to all INPs and several others by more than one INPs (Fig. 3e–h, Table 2).

**Table 1:** List of 10 top-ranked genes deregulated after individual INPs exposure

INPs	Probe name	*p*-value	Regulation	Fold change	Gene symbol
PEG-AuNPs	A_22_P00005435	0.001	Up	4.9	*lnc-DTYMK-4*
	A_22_P00015477	0.007	Up	4.2	*lnc-STAG1-2*
	A_23_P11697	0.008	Up	4.0	*HMGB4*
	A_21_P0011957	0.040	Up	3.9	*XLOC_l2_008221*
	A_24_P25544	0.015	Up	3.5	*GDNF*
	A_22_P00015552	0.003	Up	3.5	*lnc-STOM-1*
	A_33_P3243405	0.049	Down	− 3.4	*GPR182*
	A_22_P00003950	0.012	Up	3.4	*lnc-CHAD-3*
	A_22_P00015975	0.003	Up	3.3	*lnc-TELO2-3*
	A_33_P3263625	0.037	Up	3.3	*DUSP8*
Fe_3_O_4_NPs	A_22_P00003023	0.034	Up	2.9	*lnc-C7orf13-1*
	A_24_P169896	0.001	Up	2.7	*KIAA1731NL*
	A_21_P0011423	0.023	Up	2.7	*XLOC_l2_005175*
	A_22_P00011019	0.023	Up	2.6	*lnc-NT5C-1*
	A_23_P214080	4.5 x 10^-5^	Down	− 2.6	*EGR1*
	A_23_P354591	0.023	Up	2.5	*MVB12B*
	A_33_P3245679	0.027	Up	2.5	*LOC100129940*
	A_23_P106194	3.9 x 10^-5^	Down	− 2.5	*FOS*
	A_33_P3300132	4.9 x 10^-5^	Up	2.5	*FP6628*
	A_22_P00004550	0.002	Up	2.4	*lnc-CRIPT-1*
SiO_2_NPs	A_23_P81507	0.043	Down	− 4.0	*FAT2*
	A_24_P158285	0.007	Down	− 3.1	*CYLC2*
	A_21_P0012953	0.041	Down	− 2.9	*LOC102723470*
	A_33_P3379926	0.008	Down	− 2.9	*SLC17A4*
	A_21_P0005368	0.003	Up	2.8	*lnc-GARS-4*
	A_22_P00025922	0.025	Up	2.6	*LOC101929007*
	A_22_P00022177	0.003	Down	− 2.6	*lnc-UHRF2-1*
	A_23_P360302	0.048	Down	− 2.6	*GUCY2F*
	A_21_P0008347	0.017	Down	− 2.5	*lnc-FUT8-1*
	A_33_P3266429	0.023	Down	− 2.5	*SAMD13*
TiO_2_NPs	A_33_P3268863	0.027	Down	− 5.4	*C11orf44*
	A_33_P3409337	0.002	Down	− 3.6	*CCDC176*
	A_22_P00008990	0.005	Down	− 3.3	*lnc-LAMC2-1*
	A_33_P3732854	0.029	Up	3.0	*OR9H1P*
	A_24_P932736	0.032	Down	− 2.9	*HMBOX1*
	A_33_P3242323	7.6 x 10^-5^	Up	2.8	*TAF5L*
	A_22_P00005766	0.003	Up	2.6	*lnc-ENOSF1-2*
	A_22_P00015927	0.028	Down	− 2.6	*lnc-TCL1B-2*
	A_21_P0004750	0.038	Down	− 2.5	*lnc-SUPT3H-1*
	A_21_P0007854	0.036	Down	− 2.5	*lnc-HNF1A-1*

**Table 2:** List of genes significantly deregulated by all INPs

Probe name	Gene	Probe name	Gene	Probe name	Gene
Up-regulated genes			Down-regulated genes		
A_23_P116743	*LINC01089*	A_23_P315364	*CXCL2*	A_23_P108342	*ZNF571*
A_23_P376488	*TNF*	A_33_P3368358	*NEDD9*	A_23_P364544	*C12orf60*
A_32_P305888	***SH3TC2***	A_23_P379026	*GTPBP2*	A_23_P106194	***FOS***
A_23_P202837	*CCND1*	A_23_P111194	***SPDEF***	A_24_P68247	*TRIM4*
A_23_P68740	*AIRE*	A_23_P320290	*ZNF827*	A_23_P418083	*LCA5*
A_33_P3650491	*LMCD1-AS1*	A_23_P129005	*NYNRIN*	A_23_P31073	*MYB*
A_23_P19673	*SGK1*	A_33_P3244991	*lnc-EIF2AK4-4*	A_21_P0000145	*TMEM56-RWDD3*
A_23_P133408	***CSF2***	A_33_P3718269	***MIR146A***	A_24_P358425	*GPATCH11*
A_32_P377880	***GDNF***	A_22_P00005910	*LINC01252*		
A_21_P0000570	*ADORA2A-AS1*	A_33_P3214129	***LOC728061***		
A_24_P183150	*CXCL3*	A_21_P0011692	***XLOC_l2_006718***		
A_33_P3258581	*PTGES2-AS1*	A_19_P00326808	*HOTAIR*		
A_33_P3268181	***LIMS2***	A_33_P3209816	***DPY19L3***		
A_23_P155123	***CYP2D6***	A_21_P0004531	***LOC101929719***		
A_23_P383422	***NFKBID***	A_23_P71037	*IL6*		
A_33_P3232692	*IL24*	A_22_P00024437	*LOC340581*		
A_23_P73702	*MED12*	A_32_P409222	*ZNF628*		
A_22_P00012482	*LOC100506368*				

Those highlighted bold were found up- or down-regulated more or equal to 1.5-fold by at least 3 INP exposures

Among the genes up-regulated by all INPs exposure belong interleukins 24 and 6 (*IL24*, *IL6*), tumor necrosis factor (*TNF*), colony-stimulating factor 2 (*CSF2*), atypical inhibitors of NF-κB (*NFKBID*), glial cell-derived neurotrophic factor (*GDNF*), neural precursor cell expressed, developmentally down-regulated 9 (*NEDD9*), dpy-19 like C-mannosyltransferase 3 (*DPY19L3*), cyclin D1 (*CCND1*), serum/glucocorticoid regulated kinase 1 (*SGK1*), C-X-C motif chemokine ligands (*CXCL3*, *CXCL2*), cytochrome P450 family 2 subfamily D member 6 (*CYP2D6*), and TFs such as SAM pointed domain-containing ETS transcription factor (*SPDEF*), zinc finger protein 827 (*ZNF827*), zinc finger protein 628 (*ZNF628*), and autoimmune regulator (*AIRE*).

**Fig. 4:**
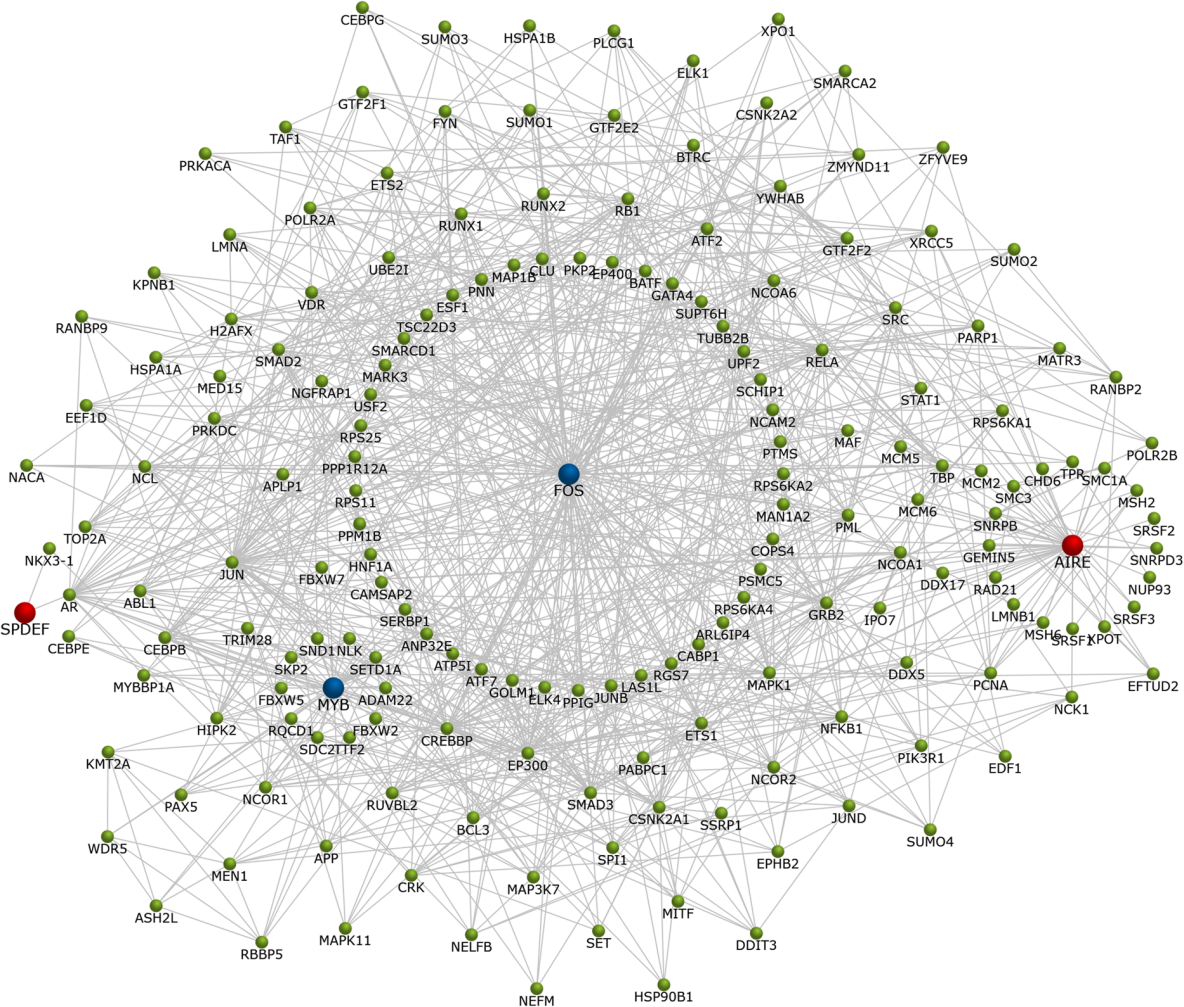
The interaction network of *SPDEF*, *AIRE*, *FOS*, and *MYB* TFs. Up-regulated TFs are highlighted by red, down-regulated by blue, interacting genes by green

Strikingly, besides zinc finger protein 571 (ZNF571), down-regulated genes were FOS proto-oncogene, AP-1 transcription factor subunit (*FOS*), and MYB proto-oncogene, transcription factor (*MYB*). The interaction network of *SPDEF*, *AIRE*, *FOS,* and *MYB* TFs is depicted in Fig. 4. Given the FOS regulatory role (Fig. 4) and since all INPs induced its down-regulation, we validated *FOS* expression changes by qRT-PCR, which confirmed microarray findings (Fig. S2).

### Pathway analysis

**Fig. 5:**
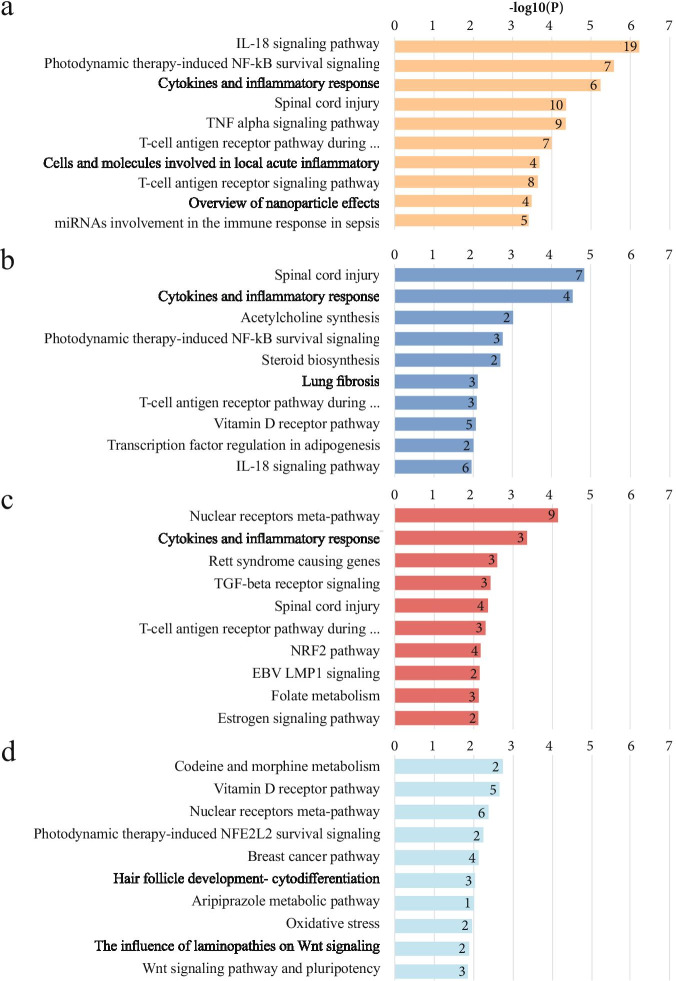
10 top-ranked metabolic pathways deregulated in cells exposed to individual INPs, namely PEG-AuNPs (**a**), Fe_3_O_4_NPs (**b**), SiO_2_NPs (**c**), and TiO_2_NPs (**d**). Over-representation analysis was performed using a list of genes with at least a 1.5-fold difference. The numbers in the bars represent the number of deregulated genes within the individual pathways

Over-representation pathway analysis using significant differentially expressed genes revealed a high number of altered pathways for all exposure types. After the restriction of the clustering analysis to the results with fold-change > 1.5, we obtained 79, 25, 34, and 33 deregulated pathways associated with PEG-AuNPs, Fe_3_O_4_NPs, SiO_2_NPs, and TiO_2_NPs exposure, respectively. The 10 top-ranked altered pathways for each INP exposure are listed in Fig. 5. Among those deregulated by all INPs were pathways related to immune responses such as inflammation (IL-18 signaling pathway, cytokines and inflammatory response, cells and molecules involved in a local acute inflammatory response, T cell receptor (TCR) signaling pathway), and other types of modulation or induction of immune responses (TNF signaling pathway, nuclear factor kappa B (NF-kB) survival signaling). While PEG-AuNPs and Fe_3_O_4_NPs exposure resulted in deregulation of photodynamic therapy-induced NF-kB survival signaling pathway, TiO_2_NPs initiated photodynamic therapy-induced NFE2L2 (NRF2) survival signaling. SiO_2_NPs and TiO_2_NPs affected nuclear receptors meta-pathway, which includes receptors controlling development and homeostasis. All significantly deregulated pathways are presented in Table S4.

### DNA methylation-mediated changes in gene expression

**Fig. 6:**
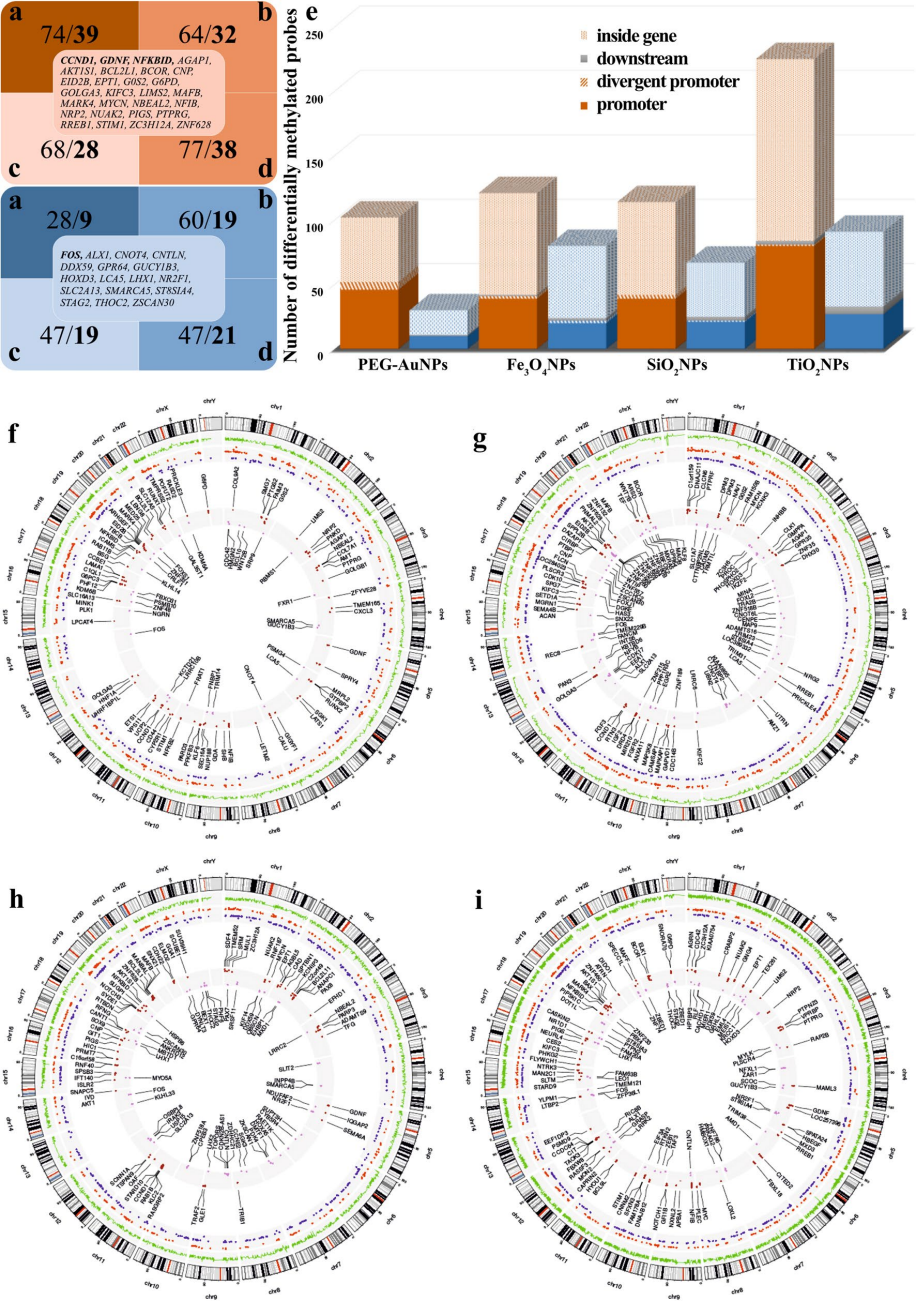
Differentially methylated probes significantly affected by individual INP treatment (**a**–**e**) and Circos graphs showing the integration of exposure-induced DNA methylation and transcriptomic changes (**f**–**i**) Hypomethylated probes/genes are highlighted by orange, hypermethylated by blue color (**a**–**d**). The number of genes with deregulated promoter methylation (both promoters and divergent promoters) correlating with gene expression after PEG-AuNPs (**a**), Fe_3_O_4_NPs (**b**), SiO_2_NPs (**c**), and TiO_2_NPs (**d**) exposure are shown in bold. Genes differentially methylated in more than one INP-exposure are listed in the middle of blue and orange squares. The location of probes/genes is shown by different patterns (**e**). Integration of DNA methylation with gene expression data for PEG-AuNPs (**f**), Fe_3_O_4_NPs (**g**), SiO_2_NPs (**h**), and TiO_2_NPs (**i**) is shown in Circos graphs. The outer circle is the chromosome idiogram of the human genome (based on G-banding, centromere highlighted in red). Green outer circos plots demonstrate the culmination of methylation data (*p* < 0.05, delta beta ± 0.15). The second plot represents significantly different up-regulated (red dots) and down-regulated (blue dots) genes (*p* < 0.05). Inner plot shows hypomethylated/up-regulated (brown) and hypermethylated/down-regulated (violet) genes.

Furthermore, we were interested in what extent were gene expression changes mediated by the deregulation of DNA methylation. We used the quantitative microarray approach to identify differentially methylated regions of INPs exposed and control TH-1 cells. DNA methylation analysis revealed different numbers of probes/genes significantly changed after individual INPs exposure (132/102, 201/124, 181/115, and 316/124 for PEG-AuNPs, Fe_3_O_4_NPs, SiO_2_NPs, and TiO_2_NPs, respectively) (Table S5), with 47%, 41.1%, 40.8%, and 47.5% of genes with inverse correlated expression (Fig. 6a–d). The majority of differentially methylated probes was located inside of the genes (52.3%, 67.2%, 63.0%, and 63.0% for PEG-AuNPs, Fe_3_O_4_NPs, SiO_2_NPs, and TiO_2_NPs, respectively), followed by gene promoters (42.4%, 29.4%, 33.1%, and 33.9% for PEG-AuNPs, Fe_3_O_4_NPs, SiO_2_NPs, and TiO_2_NPs, respectively) (Fig. 6e). The negligible number of probes (from 0.8% in PEG-AuNPs up to 2.8% in TiO_2_NPs exposed cells) was located downstream of the transcription start sites or within regions of divergent promoters (from 0.3% in TiO_2_NPs up to 4.5% in PEG-AuNPs exposed cells).

The majority of probes located in promoters were hypomethylated (82.1%, 66.1%, 65.0%, and 74.8% for PEG-AuNPs, Fe_3_O_4_NPs, SiO_2_NPs, and TiO_2_NPs, respectively). Interestingly, the *FOS* gene was hypermethylated in all exposure types. Genes *GDNF* and *NFKBID* were hypomethylated after exposure to PEG-AuNPs, SiO_2_NPs, and TiO_2_NPs, while *CCND1* after exposure to Fe_3_O_4_NPs, SiO_2_NPs, and TiO_2_NPs. The genomic locations of significantly deregulated genes are visualized using circos plots (Fig. 6f–i).

The highest number of hypomethylated/up-regulated genes after PEG-AuNPs exposure (*BCL2L1*, *LAMA1*, *RUNX1*, *PTGS2, CCND1*, *ARHGEF1*, *ETS1, NFKB2*, *SGK1*, *COL9A2*, *G6PC3*, *COL7A1*, *CD44*, *COL9A2*, *ICAM5*, *BSG*, *SHB*, *NRP2*) has functional classification in cancer-related pathways, e.g., PI3K-Akt signaling or VEGFA-VEGFR2, extracellular matrix organization, or others. Interestingly, the majority of hypermethylated/down-regulated genes (*WNT2B*, *FRAT1*, *CDC42*, *FOS*, *PSMB10*) is classified in DNA damage response, Wnt signaling, and other pathways. Although transcriptomic deregulation occurred predominantly in the immune system pathways, particularly after PEG-AuNPs exposure, we identified only several hypermethylated genes (*CDC42*, *BCL10*, *FOS*, *CRLF1*, *TRIM14*) involved in immune system regulation, namely T cell receptor signaling or cytokine signaling. Two epigenetic regulators deregulated by PEG-AuNPs exposure were lysine demethylase 6A (*KDM6A*) and its paralog 6B (*KDM6B*). KDM6A is believed to act as a tumor suppressor.

Likewise, the highest number of deregulated genes (*MAPKAP1*, *FGF3*, *AKT2*, *NRG2*, *FGFR2 IGF2*, *PTBP1*, *CCND1*, *WNT7B*) after the Fe_3_O_4_NPs exposure was identified in cancer-related pathways, among them PI3K/AKT signaling, signaling by receptor tyrosine kinases or PIP3 activated AKT signaling. Immune genes were deregulated rarely. Various cancer-related genes (*BCL2L1*, *RASGRP2*, *TRAF2*, *BCL2L11*, *CCND1*, *TFG*, *NOTCH3*, *AKT1*, *PAX8*, *AKT1*, *PARP3*, *SOX9*, *ANAPC1*, *SPTBN1*) were deregulated also by SiO_2_NPs exposure. They are involved in apoptosis regulation and TGF beta receptor signaling. IL-7 signaling, IL-2 receptor beta chain in T cell activation, or IL-3 signaling were also influenced.

TiO_2_NPs exposure affected genes (*NFIB*, *MYC*, *NOTCH1*, *BCOR*, *MAML3*, *CDC42*, *HBEGF*, *ELK1*, *TAOK3*, *PIP5K1C*, *G6PD*, *BAX*, *CES2*, *MAFF*, *AKT1S1*, *NRP2*) were over-represented in EGF-Core signaling, B cell receptor signaling, nuclear receptors meta-pathway, or VEGFA-VEGFR2 signaling. One of the hypomethylated genes was DOT1-like histone lysine methyltransferase (*DOT1L*), a histone-modifying enzyme, playing a role in several biological processes, including DNA repair, chromatin silencing, and cell cycle regulation.

Interestingly, methylation-regulated genes cover a relatively high proportion of transcription factors listed in Table S6. Gene Ontology (GO) resource was used for their precise annotation.

## Discussion

The growing number of sophisticated nanoscale materials utilized in medicine and everyday products highlights the need for new strategies to comprehensively evaluate their biosafety for human health. The involvement of “omics” technologies in the risk assessment of INPs contributes to a better understanding of the molecular changes occurring in cells/tissues in response to INPs’ exposure, allowing the prediction of their mechanism of action and potential toxicity (Nymark et al. 2020). One of the advantages is their ability to detect new targets and adverse outcome pathways at low but physiologically more relevant NPs’ concentrations at which no phenotypic changes can be revealed using conventional test models. In line with this strategy, we used the whole transcriptome and genome-wide methylome analysis to comprehensively evaluate the potential adverse health effects of selected INPs. To the best of our knowledge, this is the first study providing a comprehensive assessment of DNA methylation-mediated transcriptomic changes allowing for a more relevant interpretation of INPs-induced functional effects. Interestingly, despite the INP-specific pattern of gene expression were found in our study, genes coding for long non-coding RNAs (lncRNA) dominated the list of 10 top-ranked genes in all treatments. LncRNAs play a pivotal role in regulating the genome at different levels, including the epigenetic processes, nuclear architecture, and gene expression. They act as nucleo- or cytoplasmic scaffolds, modulate mRNA splicing, stability, control translation, and post-translational modifications and interfere with signaling pathways (Statello et al. 2021). Their highly tissue-specific expression pattern indicates that lncRNA transcription is intimately linked with complex cellular biology, development, and health. Dysregulation of lncRNAs expression is closely associated with many diseases, including cancer. Despite the vast amount of annotated human lncRNAs (> 215,000) (Ignarski et al. 2019), they remain to be functionally characterized because lncRNAs have only been discovered in the last decade. Notably, all INPs significantly up-regulated HOX antisense intergenic RNA (*HOTAIR*) expression in TH-1 cells. This recently discovered lncRNA has been associated with tumorigenesis, invasion, metastasis, and cancer drug resistance (Tang and Hann 2018).

The vast majority of transcriptomic studies published to date revealed deregulation of gene expression and pathways associated with immune response and inflammation after short-term (mainly 24 h) exposure to INPs (SiO_2_NPs, iron oxide NPs, TiO_2_NPs) or carbon nanotubes (reviewed in (Pogribna and Hammons 2021)). In agreement with these findings, we also identified transcriptomic changes in immune and inflammation pathways (*IL24*, *IL6*, *TNF*, *CSF2*, *NFKBID*, *CXCL3*, *CXCL2*). Remarkably, Gliga et al. (2018) reported also deregulation of genes associated with fibrosis and epithelial-mesenchymal transition (EMT) pathway in human lung BEAS-2B cells after long-term (6 weeks) exposure to silver NPs (AgNPs).

Genes up-regulated by all studied INPs were also two zinc finger proteins (*ZNF827* and *ZNF628*) and TFs *SPDEF* and *AIRE*. Zinc finger proteins, as the largest family of sequence-specific DNA binding proteins, are involved in the regulation of numerous cellular processes, including transcription, signal transduction, DNA repair, cell migration, autophagy, or chromatin remodeling. There is growing evidence indicating the potential role of ZNF proteins in several diseases, including cancer progression and metastasis. However, the same ZNF protein can act as an oncogene or tumor suppressor depending on the regulation level (Jen and Wang 2016). Interestingly, *SPDF* overexpression was shown to suppress intrinsic, innate immune signaling, thereby inhibiting inflammation (Korfhagen et al. 2012). AIRE is a central protein in maintaining immune tolerance (Huoh et al. 2020), whose expression was identified at both mRNA and protein levels in renal epithelial cells of tubules and podocytes (Lovewell and Tazi-Ahnini 2011). Besides possible adverse effects, its up-regulation might indicate the physiological response of the immune system to the foreign substance. Our hypothesis supports the fact that interleukin-6 (IL-6) is directly regulated by AIRE, and a significant increase in IL-6 level was detected in AIRE-overexpressed cells (Kalra et al. 2018). In contrast to *AIRE* and *SPDEF*, all INPs significantly down-regulated the *FOS* expression in TH-1 cells. As a subunit of AP-1, *FOS* is involved in the regulation of a wide range of cellular processes such as proliferation, differentiation, cell death, or immune response. Its deregulation is associated with various pathological conditions, including the transformation and progression of cancer. Interestingly, recent research on chronic kidney disease (CKD) has identified *FOS* as one of the five hub genes that might play critical roles in regulating the development of CKD (Zhou et al. 2018a). RNA-seq data analysis showed a significant down-regulation of the *FOS* gene along with others in tubulointerstitial samples from patients with CKD (Guo et al. 2019). However, the molecular mechanisms underlying CKD, a complex heterogeneous disease, are poorly understood. Nevertheless, the capacity of INPs to down-regulate *FOS* due to their long-term accumulation in kidney tissue might indicate their potential hazard for human health although further studies are needed to confirm their role in CKD pathogenesis.

Surprisingly, compared to gene expression data, a relatively high proportion of genes identified to be differentially methylated after INPs exposure were TFs. Among them, only *FOS* was hypermethylated in all INPs exposures, which correlated with its transcriptomic down-regulation. *CCND1*, *GDNF*, and *NFKBID* genes were hypomethylated/up-regulated after exposure to three INPs. These TFs are involved in essential renal functions such as regulation of cell cycle, cell growth and differentiation, inflammation, and immune function (Uddin et al. 2019).

In general, the number of studies providing whole-genome methylation data or data reporting long-term exposure effects is limited. Their vast majority comes from in vitro short-term treatment using different cell types and INPs differing in physicochemical characteristics. For example, a correlation between an increase in global DNA methylation and the expression of DNA methyltransferases was observed in normal human fibroblasts but not melanoma A375 cells after 24 h exposure to AuNPs (Patil et al. 2019). In contrast, no change in global DNA methylation was found in HaCaT and HEK293 cells (Sooklert et al. 2019), human breast SK-BR-3 cells (Smolkova et al. 2016), or human hepatoma HepG2 cells (Brzóska et al. 2019) after the short-term exposure to AuNPs. In our study, we identified 102 genes differentially methylated; promoter methylation was present in 48 of them, including 11 TFs. Among them, for example, hepatocyte nuclear factor 1-alpha (*HNF1A*) required for the expression of drug transporters in the kidney was hypomethylated (Martovetsky et al. 2013). PEG-AuNPs exposure also caused hypomethylation in RUNX1 family transcription factor 1 (*RUNX1*) and 2 (*RUNX2*) genes. Overexpression of *RUNX1* has been shown to promote the expression of EMT marker genes in renal tubular epithelial cells and renal fibrosis (Zhou et al. 2018b). *RUNX1* and *RUNX2* genes might be also involved in kidney cancer (Rooney et al. 2020).

The only study published to date did not find any changes in global methylation after short-term exposure of HepG2 cells to Fe_3_O_4_NPs (Brzóska et al. 2019). In contrast, we identified 201 differentially methylated regions (DMRs) covering 124 annotated genes, promoter methylation affected in 51 of them, including 19 TFs in TH-1 cells. Fe_3_O_4_NPs induced hypomethylation, for example, in MYCN proto-oncogene, BHLH transcription factor (*MYCN*), and MAF BZIP transcription factor B (*MAFB*) genes. These genes encode TFs that regulate cell growth, proliferation, and differentiation of the podocytes, the proximal tubules, and hepatocytes (Tsuchiya 2015). On the other hand, Fe_3_O_4_NPs exposure resulted in hypermethylation of the early growth response 2 (*EGR2*) gene playing a crucial role in immune system regulation and B and T cell activation (Taefehshokr et al. 2017).

All in vitro studies published to date identified a global decrease of DNA methylation in different cell lines following the short-term (24–48 h) exposure to SiO_2_NPs (Sooklert et al. 2019). However, gene-specific changes have been studied rarely. The impact of repeated long-term exposure (30 passages) to SiO_2_NPs was assessed on BEAS-2B cells. Genome-wide analysis identified a significant predominance of hypermethylation (1973 CpG loci) over hypomethylation (223 loci); however, relevant transcriptomic changes were not assessed (Zou et al. 2016). In our study, we identified 181 DMRs negatively correlating with changes in gene expression. DMRs covered 115 genes, of which 47 were located in the gene promoters, including 8 TFs. SiO_2_NPs, similarly to Fe_3_O_4_NPs induced hypomethylation/up-regulation of *MYCN* and *MAFB* genes. Additionally, hypomethylated/up-regulated was also SRY-Box transcription factor 9 (SOX9), which acts as a regulator of cell differentiation during the development (Menzel-Severing et al. 2018). Gene expression profiles in renal biopsies from CKD patients showed a significant relationship of increased SOX9 expression with tubulointerstitial fibrosis and tubular cell damage (Nakagawa et al. 2015). SiO_2_NPs also induced hypermethylation/down-regulation of MAX dimerization protein 1 (*MXD1*), homeobox A5 (*HOXA5*), and cyclin D binding Myb-like transcription factor 1 (*DMTF1*) genes. Recent epigenome-wide methylation analysis has revealed that DNA methylation-dependent *HOXA5* repression could contribute to pathologic tissue remodeling seen in CKD-related cardiovascular disease (Dritsoula et al. 2020). *DMTF1* functions as a tumor suppressor inducing cell growth arrest or apoptosis.

Short-term exposure (24–72 h) to TiO_2_NPs resulted in decreased global DNA methylation and altered expression levels of methylation-related genes and proteins in various cell lines (Pogribna et al. 2020). However, no data on long-term exposure or large-scale epigenomic effects are available so far. Interestingly, in our study, TiO_2_NPs induced the highest number of DNA methylation changes in comparison to PEG-AuNPs, Fe_3_O_4_NPs, and SiO_2_NPs. We found differentially methylated 124 genes, with methylation changes also in promoter region in 59 of them, including 9 TFs. Hypomethylation MAF transcription factor F (*MAFF*) belongs to the important transcriptional regulators of the stress response and detoxification pathways (Katsuoka and Yamamoto 2016). Its up-regulation might be closely related to the accumulation of TiO_2_NPs inside the cells. Other hypomethylated genes were nuclear receptor subfamily 1 group D member 1 (NR1D1) and ETS transcription factor (*ELK1*). *NR1D1* and *ELK1* gene products regulate the inflammatory responses (Liu et al. 2020). TiO_2_NPs-mediated hypermethylation was detected in grainyhead-like transcription factor 1 (*GRHL1*) and zinc finger E-box-binding homeobox 1 (*ZEB1*) genes. *GRHL1* is a tumor suppressor. High levels of the *GRHL1* expression correlate with a favorable prognosis for neuroblastoma cancer patients (Mlacki et al. 2015), while its down-regulation might promote cell proliferation. ZEB1 is a TF essential to the physiological processes of differentiation, cell growth, and cell death (Zhang et al. 2019). Down-regulation of ZEB1 resulted in an inhibitory effect on the invasive and metastatic potential of epithelial ovarian cancer in vitro and in vivo models by blocking the EMT process (Chen et al. 2013).

In conclusion, chronic exposure of TH-1 cells to low sub-cytotoxic INPs’ concentrations indicated their potential epigenetic toxicity. Our results highlight the need for a more comprehensive investigation of the possible adverse effects of INPs, with particular attention on the epigenetic regulatory mechanisms after chronic exposure. The benefit of INPs in biomedicine is considerable, namely in terms of diagnostics and therapy. New omics-based risk assessment approaches of INPs can help better elucidate their mechanism of action and contribute to identifying new, more specific biomarkers of exposure.

### Supplementary Information

ESM 1 (DOCX 13 kb)

ESM 2 (DOCX 13 kb)

ESM 3 (XLSX 562 kb)

ESM 4 (XLSX 26 kb)

ESM 5 (XLSX 161 kb)

ESM 6 (DOCX 13 kb)

ESM 7 (DOCX 31 kb)

ESM 8 (DOCX 139 kb)

## References

[CR1] Bayda S, Hadla M, Palazzolo S, Riello P, Corona G, Toffoli G (2018). Inorganic nanoparticles for cancer therapy: a transition from lab to clinic. Curr Med Chem.

[CR2] Brzóska K, Gradzka I, Kruszewski M. Silver, gold, and iron oxide nanoparticles alter miRNA expression but do not affect DNA methylation in HepG2 cells. Materials (Basel). 2019;12(7). 10.3390/ma12071038. 10.3390/ma12071038 PMC6479689 30934809

[CR3] Buocikova V, Rios-Mondragon I, Pilalis E, Chatziioannou A, Miklikova S, Mego M (2020). Epigenetics in breast cancer therapy—new strategies and future nanomedicine perspectives. Cancers (Basel).

[CR4] Chen D, Wang J, Zhang Y, Chen J, Yang C, Cao W (2013). Effect of down-regulated transcriptional repressor ZEB1 on the epithelial-mesenchymal transition of ovarian cancer cells. Int J Gynecol Cancer.

[CR5] Dadfar SM, Roemhild K, Drude NI, von Stillfried S, Knüchel R, Kiessling F (2019). Iron oxide nanoparticles: diagnostic, therapeutic and theranostic applications. Adv Drug Deliv Rev.

[CR6] Dritsoula A, Kislikova M, Oomatia A, Webster AP, Beck S, Ponticos M, et al. “Epigenome-wide methylation profile of chronic kidney disease-derived arterial DNA uncovers novel pathways in disease-associated cardiovascular pathology.” Epigenetics. Taylor & Francis; 2020;1–11. 10.1080/15592294.2020.1819666 10.1080/15592294.2020.1819666 PMC8216178 32930636

[CR7] Dusinska M, Tulinska J, El Yamani N, Kuricova M, Liskova A, Rollerova E (2017). Immunotoxicity, genotoxicity and epigenetic toxicity of nanomaterials: New strategies for toxicity testing?. Food Chem Toxicol.

[CR8] Gliga AR, Di Bucchianico S, Lindvall J, Fadeel B, Karlsson HL (2018). RNA-sequencing reveals long-term effects of silver nanoparticles on human lung cells. Sci Rep Springer US.

[CR9] Guo Y, Ma J, Xiao L, Fang J, Li G, Zhang L (2019). Identification of key pathways and genes in different types of chronic kidney disease based on WGCNA. Mol Med Rep.

[CR10] Huoh YS, Wu B, Park S, Yang D, Bansal K, Greenwald E (2020). Dual functions of Aire CARD multimerization in the transcriptional regulation of T cell tolerance. Nat Commun Springer US.

[CR11] Ignarski M, Islam R, Müller RU. Long non-coding RNAs in kidney disease. Int J Mol Sci. 2019;20(13). 10.3390/ijms20133276. 10.3390/ijms20133276 PMC6650856 31277300

[CR12] Jafari S, Derakhshankhah H, Alaei L, Fattahi A, Varnamkhasti BS, Saboury AA (2019). Mesoporous silica nanoparticles for therapeutic/diagnostic applications. Biomed Pharmacother Elsevier.

[CR13] Jafari S, Mahyad B, Hashemzadeh H, Janfaza S, Gholikhani T, Tayebi L. Biomedical applications of TiO 2 nanostructures : recent advances. 2020;3447–70. 10.2147/IJN.S249441. 10.2147/IJN.S249441 PMC7234979 32523343

[CR14] Jen J, Wang YC (2016). Zinc finger proteins in cancer progression. J Biomed Sci.

[CR15] Kalra R, Bhagyaraj E, Tiwari D, Nanduri R, Chacko AP, Jain M, et al. AIRE promotes androgen-independent prostate cancer by directly regulating IL-6 and modulating tumor microenvironment. Oncogenesis Springer US. 2018;7(5). 10.1038/s41389-018-0053-7. 10.1038/s41389-018-0053-7 PMC5968032 29795364

[CR16] Katsuoka F, Yamamoto M (2016). Small Maf proteins (MafF, MafG, MafK): History, structure and function. Gene.

[CR17] Korfhagen TR, Kitzmiller J, Chen G, Sridharan A, Haitchi HM, Hegde RS (2012). SAM-pointed domain ETS factor mediates epithelial cell-intrinsic innate immune signaling during airway mucous metaplasia. Proc Natl Acad Sci. U S A.

[CR18] Kurtzeborn K, Cebrian C, Kuure S (2018). Regulation of renal differentiation by trophic factors. Front Physiol.

[CR19] Liou SH, Te WW, Liao HY, Chen CY, Tsai CY, Jung WT (2017). Global DNA methylation and oxidative stress biomarkers in workers exposed to metal oxide nanoparticles. J Hazard Mater Elsevier BV.

[CR20] Liu H, Zhu Y, Gao Y, Qi D, Zhao L, Zhao L, et al. NR1D1 modulates synovial inflammation and bone destruction in rheumatoid arthritis. Cell Death Dis Springer US. 2020;11(2). 10.1038/s41419-020-2314-6. 10.1038/s41419-020-2314-6 PMC7028921 32071294

[CR21] Lovewell T, Tazi-Ahnini R (2011). Models to explore the molecular function and regulation of AIRE. Egypt. J. Med. Hum. Genet.

[CR22] Ma Y, Guo Y, Ye H, Huang K, Lv Z, Ke Y (2019). Different effects of titanium dioxide nanoparticles instillation in young and adult mice on DNA methylation related with lung inflammation and fibrosis. Ecotoxicol Environ Saf.

[CR23] Martovetsky G, Tee JB, Nigam SK (2013). Hepatocyte nuclear factors 4α and 1α regulate kidney developmental expression of drug-metabolizing enzymes and drug transporters. Mol Pharmacol.

[CR24] Menzel-Severing J, Zenkel M, Polisetti N, Sock E, Wegner M, Kruse FE (2018). Transcription factor profiling identifies Sox9 as regulator of proliferation and differentiation in corneal epithelial stem/progenitor cells. Sci Rep.

[CR25] Mlacki M, Kikulska A, Krzywinska E, Pawlak M, Wilanowski T (2015). Recent discoveries concerning the involvement of transcription factors from the Grainyhead-like family in cancer. Exp Biol Med.

[CR26] Nakagawa S, Nishihara K, Miyata H, Shinke H, Tomita E, Kajiwara M (2015). Molecular markers of tubulointerstitial fibrosis and tubular cell damage in patients with chronic kidney disease. PLoS One.

[CR27] Neri F, Rapelli S, Krepelova A, Incarnato D, Parlato C, Basile G (2017). Intragenic DNA methylation prevents spurious transcription initiation. Nature. Nature Publishing Group.

[CR28] Nymark P, Bakker M, Dekkers S, Franken R, Fransman W, García-Bilbao A (2020). Toward rigorous materials production: new approach methodologies have extensive potential to improve current safety assessment practices. Small.

[CR29] Ognik K, Cholewińska E, Juśkiewicz J, Zduńczyk Z, Tutaj K, Szlązak R (2019). The effect of copper nanoparticles and copper (II) salt on redox reactions and epigenetic changes in a rat model. J Anim Physiol Anim Nutr (Berl).

[CR30] Patil YM, Rajpathak SN, Deobagkar DD (2019). Characterization and DNA methylation modulatory activity of gold nanoparticles synthesized by Pseudoalteromonas strain. J Biosci.

[CR31] Pogribna M, Hammons G. Epigenetic effects of nanomaterials and nanoparticles. J Nanobiotechnol BioMed Central. 2021;19(1). 10.1186/s12951-020-00740-0. 10.1186/s12951-020-00740-0 PMC7789336 33407537

[CR32] Pogribna M, Koonce NA, Mathew A, Word B, Patri AK, Lyn-Cook B (2020). Effect of titanium dioxide nanoparticles on DNA methylation in multiple human cell lines. Nanotoxicology Taylor & Francis.

[CR33] Ray PD, Yosim A, Fry RC. Incorporating epigenetic data into the risk assessment process for the toxic metals arsenic, cadmium, chromium, lead, and mercury: strategies and challenges. Front Genet 2014;5(JUL):1–26. 10.3389/fgene.2014.00201. 10.3389/fgene.2014.00201 PMC4100550 25076963

[CR34] Rooney N, Mason SM, McDonald LM, Däbritz JH, Campbell KJ, Hedley A (2020). RUNX1 is a driver of renal cell carcinoma correlating with clinical outcome. Cancer Res.

[CR35] Rossnerova A, Honkova K, Pelclova D, Zdimal V, Hubacek JA, Chvojkova I, et al. DNA methylation profiles in a group of workers occupationally exposed to nanoparticles. Int J Mol Sci 2020;21(7). 10.3390/ijms21072420. 10.3390/ijms21072420 PMC7177382 32244494

[CR36] Singh P, Pandit S, Mokkapati VRSS, Garg A, Ravikumar V, Mijakovic I. Gold nanoparticles in diagnostics and therapeutics for human cancer. Int. J. Mol. Sci. 2018;19(7). 10.3390/ijms19071979. 10.3390/ijms19071979 PMC6073740 29986450

[CR37] Smolkova B, Miklikova S, Horvathova Kajabova V, Babelova A, El Yamani N, Zduriencikova M (2016). Global and gene specific DNA methylation in breast cancer cells was not affected during epithelial-to-mesenchymal transition in vitro B. Neoplasma.

[CR38] Sooklert K, Nilyai S, Rojanathanes R, Jindatip D, Sae-Liang N, Kitkumthorn N (2019). N-acetylcysteine reverses the decrease of DNA methylation status caused by engineered gold, silicon, and chitosan nanoparticles. Int J Nanomed.

[CR39] Sramkova M, Kozics K, Masanova V, Uhnakova I, Razga F, Nemethova V (2019). Kidney nanotoxicity studied in human renal proximal tubule epithelial cell line TH1. Mutat Res - Genet Toxicol Environ Mutagen Elsevier.

[CR40] Statello L, Guo CJ, Chen LL, Huarte M (2021). Gene regulation by long non-coding RNAs and its biological functions. Nat Rev Mol Cell Biol Springer US.

[CR41] Tabish AM, Poels K, Byun HM, Luyts K, Baccarelli AA, Martens J (2017). Changes in DNA methylation in mouse lungs after a single intra-tracheal administration of nanomaterials. PLoS One.

[CR42] Taefehshokr S, Key YA, Khakpour M, Dadebighlu P, Oveisi A (2017). Early growth response 2 and Egr3 are unique regulators in immune system. Cent Eur J Immunol.

[CR43] Tang Q, Hann SS (2018). HOTAIR: An oncogenic long non-coding RNA in human cancer. Cell Physiol Biochem.

[CR44] Tsuchiya M (2015). Transcriptional factors, Mafs and their biological roles. World J Diabetes.

[CR45] Uddin N, Minhas K, Abdul-Ghafar J, Ahmed A, Ahmad Z (2019). Expression of cyclin D1 in clear cell sarcoma of kidney. Is it useful in differentiating it from its histological mimics?. Diagn Pathol.

[CR46] Yamani NN, Collins AR, Rundén-Pran E, Fjellsbø LM, Shaposhnikov S, Zienolddiny S (2017). In vitro genotoxicity testing of four reference metal nanomaterials, titanium dioxide, zinc oxide, cerium oxide and silver: Towards reliable hazard assessment. Mutagenesis.

[CR47] Zhang Y, Xu L, Li A, Han X (2019). The roles of ZEB1 in tumorigenic progression and epigenetic modifications. Biomed Pharmacother Elsevier.

[CR48] Zhou LT, Qiu S, Lv LL, Li ZL, Liu H, Tang RN (2018). Integrative bioinformatics analysis provides insight into the molecular mechanisms of chronic kidney disease. Kidney Blood Press Res.

[CR49] Zhou T, Luo M, Cai W, Zhou S, Feng D, Xu C (2018). Runt-Related Transcription Factor 1 (RUNX1) Promotes TGF-β-Induced Renal Tubular Epithelial-to-Mesenchymal Transition (EMT) and Renal Fibrosis through the PI3K Subunit p110δ. EBioMedicine.

[CR50] Zou Y, Li Q, Jiang L, Guo C, Li Y, Yu Y (2016). DNA Hypermethylation of CREB3L1 and Bcl-2 Associated with the Mitochondrial-Mediated Apoptosis via PI3K/Akt Pathway in Human BEAS-2B Cells Exposure to Silica Nanoparticles. PLoS One.

