# Supplementary material for: Genome-wide DNA methylome and transcriptome changes induced by inorganic nanoparticles in human kidney cells after chronic exposure
Source: Cell Biol Toxicol. 2022 Jan 1;39(5):1939–56. doi: 10.1007/s10565-021-09680-3 (PMC10547624; doi:10.1007/s10565-021-09680-3)
Supplement: Supplementary file 1 — (DOCX 13 kb) [file 10565_2021_9680_MOESM1_ESM.docx]

Table S1 Primer sequences for quantitative qRT-PCR

| Gene |  | Sequence |
| --- | --- | --- |
| *HPRT1* | RT-F | 5′-CATTGTAGCCCTCTGTGTGC-3′ |
|  | RT-R | 5′-CTTTTATGTCCCCTGTTGACTG-3′ |
| *SDHA* | RT-F | 5′-AGAACATCGGAACTGCGACT-3′ |
|  | RT-R | 5′-CGAACGTCTTCAGGTGCTTT-3′ |
| *TBP* | RT-F | 5′-TGAGGATAAGAGAGCCACGAA-3′ |
|  | RT-R | 5′-AGTCCAAGAACTTAGCTGGAAA-3′ |
| *FOS* | RT-F | 5′-GGTGGAACAGTTATCTCCAGAA-3′ |
|  | RT-R | 5′-TCTGTCTCCGCTTGGAGTG-3′ |
