# Supplementary material for: Genome-wide DNA methylome and transcriptome changes induced by inorganic nanoparticles in human kidney cells after chronic exposure
Source: Cell Biol Toxicol. 2022 Jan 1;39(5):1939–56. doi: 10.1007/s10565-021-09680-3 (PMC10547624; doi:10.1007/s10565-021-09680-3)
Supplement: Supplementary file 2 — (DOCX 13 kb) [file 10565_2021_9680_MOESM2_ESM.docx]

**Table S2.** The numbers of significantly deregulated entities in TH-1 cells exposed to INPs compared to controls.

|  | PEG-AuNPs | Fe_3_O_4_NPs | SiO_2_NPs | TiO_2_NPs |
| --- | --- | --- | --- | --- |
| All INPs | 1232 | 1534 | 1314 | 1537 |
| ≥ 1.5 fold change | 1011 | 392 | 318 | 286 |
| ≥ 2 fold change | 190 | 43 | 45 | 45 |
