# Supplementary material for: Genome-wide DNA methylome and transcriptome changes induced by inorganic nanoparticles in human kidney cells after chronic exposure
Source: Cell Biol Toxicol. 2022 Jan 1;39(5):1939–56. doi: 10.1007/s10565-021-09680-3 (PMC10547624; doi:10.1007/s10565-021-09680-3)
Supplement: Supplementary file 6 — (DOCX 13 kb) [file 10565_2021_9680_MOESM6_ESM.docx]

**Table S6.** List of transcription factors (GO annotations), deregulated after individual INPs exposure.

| INPs | ***Promoter/ divergent promoter / downstream** | **Inside** |
| --- | --- | --- |
| PEG-AuNPs | *NFKB2, HNF1A, RUNX1,* ***FOS, ZNF48, ZNF331, FOXS1*** | *RUNX2, NFIB, KLF6, ETS1* |
| Fe_3_O_4_NPs | *MYCN, ZNF628, MAFB,* ***IKZF2, ZNF518B, EGR2, ALX1, ZNF557*** | *ZNF35, TEF,* ***FOXL2, ZNF215, EEA1, NFYB, FOS, ZSCAN30, ZNF284, ZNF280B, KLF8*** |
| SiO_2_NPs | *MYCN, SOX9, ZNF628, MAFB,* ***MXD1, HOXA5, DMTF1, ZKSCAN1*** | *HIC1,* ***NR2F1, TOPORS, LHX2, ZNF518A, FOS, LHX1, ZSCAN30*** |
| TiO_2_NPs | *NR1D1, ZNF460, MAFF, ELK1,* ***GRHL1, MEIS1, ZEB1, FOS, LHX1*** | *MXD3, MYC, NFIB, GFI1B,* ***NFXL1, NR2F1, ZNF786, ALX1, PBX4*** |

Bold highlighted are hypermethylated/down-regulated TFs. *Those with at least one probe located in a given region
