# Supplementary material for: Genome-wide DNA methylome and transcriptome changes induced by inorganic nanoparticles in human kidney cells after chronic exposure
Source: Cell Biol Toxicol. 2022 Jan 1;39(5):1939–56. doi: 10.1007/s10565-021-09680-3 (PMC10547624; doi:10.1007/s10565-021-09680-3)
Supplement: Supplementary file 7 — (DOCX 31 kb) [file 10565_2021_9680_MOESM7_ESM.docx]

The impact of solvents (TMAOH and Mili Q water) on viability and apoptosis of TH-1 cells.

The impact of solvents (TMAOH and Mili-Q water) on proliferation activity of TH-1 cells

Fig. S1
