# Supplementary figures and images for: Genome-wide DNA methylome and transcriptome changes induced by inorganic nanoparticles in human kidney cells after chronic exposure
Source: Cell Biol Toxicol. 2022 Jan 1;39(5):1939–56. doi: 10.1007/s10565-021-09680-3 (PMC10547624; doi:10.1007/s10565-021-09680-3)

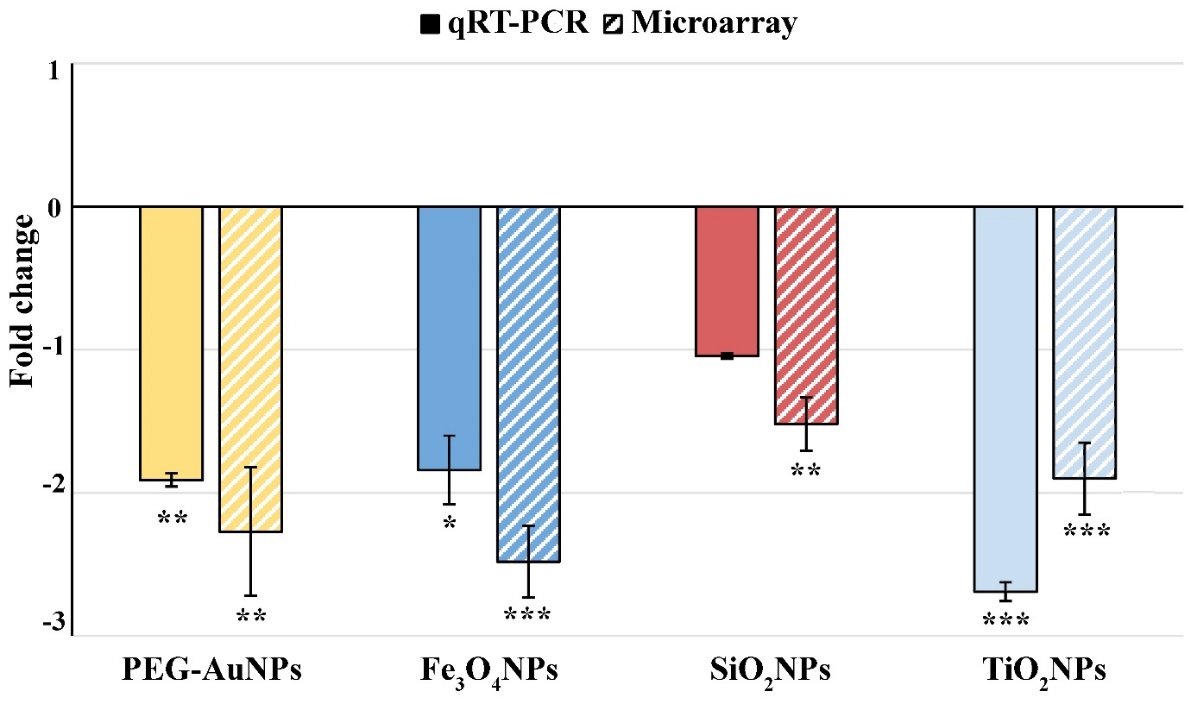


**Fig. S2** Validation of *FOS* expression in TH-1 cells exposed to INPs for 7 days by qRT-PCR.

Supplement: Supplementary file 8 — (DOCX 139 kb) [file 10565_2021_9680_MOESM8_ESM.docx]
